# The Narrow House

**Published:** 1862-03

**Authors:** 


**THE NARROW HOUSE.**—The promenader on glorious Broadway has many a time noticed a little, low, dingy-looking brick house, so contracted in front, that the show-window leaves so "strait" a door for entrance, that a "skeleton" must be compressed, or it could never cross its threshold. Yet very few half-hours pass in the day-time, in which some man or boy, woman or maid, does not seek admittance. The fact is, if the little old shanty had not been there so long, it would not have been noticed at all by the habitués of the street. Not one in a thousand would care to take a second look at it, unless in special search of something in the line of business to which it seems appropriated. That any one lived there at all, except the "man and boy," always on hand, or the bare conception that a family lived there beside, would certainly never enter the imagination of two in a million. And yet a family does live up-stairs, a family consisting of one old woman and the man in the shop; they have lived there a quarter of a century; and more, they raised a family of children there, and all of them have been married long enough to have children of their own. More than this, the old man makes all his wares in the rear, then brings them in front, to expose for sale in the one window of his "narrow house." There were three children, daughters, who went out, and returned from school, for a long series of years, and so did the music-teacher, for there was cultivation there; but there were no servants, no cooks, chambermaids, or waiters; they never had any, never wanted any; they did their own work, and do it now; were always happy, and are happy now. They all waited on themselves and on one another, and are, to-day, models of self-reliance and personal independence. The "girls" never "went into society;" society never knew them, never wanted to know them, and never invited them; they lived in such a "narrow house." But it was their own, and had no mortgage on it, as had the "forty-foot" mansion fronting on Union Park, which was sold for taxes last year.

So, being excluded from society, by the simple process of omission, they made a society of themselves, and became wise, contented, and happy; they had other things to do beside laying plans to climb among those who never could see them. In this way, they grew up without the mortifications inseparable from both society and servants, as wide as they are apart; as a consequence, there was so much of sunshine in the faces of these three girls, and such a native dignity and independence of manner, that three substantial young men, in their own sphere of life, found them out, without the use of a microscope. And just look now, how the "old lady" manages it. A downright philosopher is the mistress of the "Narrow House." She insists upon it, that a large house for two old people is like an empty barn; that big houses entail trouble, invite loungers, and keep things at a melancholy and freezing distance; and that her own cozy, little, clean rooms, and "Hubby" beside her, when the day's work is done, have more comfort in them than a palace. She further insists on it, that servants are more trouble than they are worth; that their selfishness eats out one's benevolence; their reckless wastefulness and their little thieveries, their willful ignorance and their habitual blarney and deception, never can fail to sour the temper, ruffle the feelings, and be an everlasting source of annoyance to the household. Still, she insists that she is getting old now, and wants to consult her own ease and comfort, and that while she is always glad to know that her children and grand-children are well and happy, she does not want them to be popping in upon her at any and all times; hence, one day in the week she allots to receiving her company, and on that one day in a week, rain or shine, the three daughters may be seen entering the "Narrow House," leading by the hand a sweet, chubby child or two; and there, all at home, with no stranger eyes to mar the joy, and with mutual affection to warm the heart, there is an elysium below. At dusk, "Father" comes up, and soon thereafter, the three sons-in-law, to make merry till the hour of retiring. It is remarked of the "old man," that he never made a note, never asked a bank accommodation, never failed, never suspended, never even solicited an extension; and more, he never joined any "society," except a Christian church; he says that includes all societies which have good for their object, and that, to join any other, is an implication; that being a Christian does not meet all the wants of humanity; hence, he can not practically make that admission. He was never run for office; he never entered a grocery, never "treats," never was treated. He takes no interest in politics, beyond that of making it his duty *always* to vote for reputable and educated men. The old man is rich. His sons-in-law are thrifty men, and do not want his aid. The manner in which he intends to dispose of his estate is rather peculiar; but there is a ring of wisdom, humanity, and patriotism in it, which may well be imitated. The interest only of each share is to be used by the daughters; the principal to fall to the grand-children at the mother's death; if no grand-children survive, then it becomes the property of certain charitable institutions. No interest can be drawn without the daughters' written order, and can never be drawn in advance. In this manner, he hopes to prevent any child of his coming to want, whatever may be the reverses of their husbands, the property being inalienable under any circumstances, and can not be jeopardized by any act of the daughter, making outside pressure wholly objectless.

From this narration, the thoughtful reader may gather that there is great safety in a quiet, unpretending, and unostentatious life; that families who live to themselves, and depend on themselves and one another, may be useful, prosperous, and happy; and that, although they may not become the ornaments of society, they are at once its pillars and its chief foundation-stone, while their influence for good passes to the second, and even third generation, even in their own lifetime.

